# Transcript- and protein-level analyses of the response of human eosinophils to glucocorticoids

**DOI:** 10.1038/sdata.2018.275

**Published:** 2018-12-04

**Authors:** Manasi Gadkari, Michelle A. Makiya, Fanny Legrand, Kindra Stokes, Thomas Brown, Katherine Howe, Paneez Khoury, Zonghui Hu, Amy Klion, Luis M. Franco

**Affiliations:** 1 Laboratory of Immune System Biology, National Institute of Allergy and Infectious Diseases, National Institutes of Health, 4 Memorial Drive, Building 4, Room 138, Bethesda, MD 20892, USA; 2 Human Eosinophil Section, Laboratory of Parasitic Diseases. National Institute of Allergy and Infectious Diseases, National Institutes of Health, 4 Memorial Drive, Building 4, Room B1-28, Bethesda, MD 20892, USA; 3 Clinical Parasitology Unit, Laboratory of Parasitic Diseases, National Institute of Allergy and Infectious Diseases, National Institutes of Health. 10 Center Drive, Building 10, Room 6D44, Bethesda, MD 20892, USA; 4 Laboratory of Clinical Immunology and Microbiology. National Institute of Allergy and Infectious Diseases, National Institutes of Health, 10 Center Drive, Building 10, Room 12C103, Bethesda, MD 20892, USA; 5 Bioinformatics Research Branch, National Institute of Allergy and Infectious Diseases, National Institutes of Health, 5601 Fishers Lane, Rockville, MD 20892, USA

**Keywords:** Translational research, Translational immunology, RNA sequencing, Therapeutics

## Abstract

Glucocorticoids are first-line agents for the treatment of many eosinophil-associated disorders; however, their effects on human eosinophils remain poorly understood. To gain an unbiased, genome-wide view of the early transcriptional effects of glucocorticoids on human eosinophils *in vivo*, RNA sequencing was performed on purified blood eosinophils obtained before and 30, 60, and 120 minutes after administration of a single dose of oral prednisone (1 mg/kg) to three unrelated healthy subjects with hypereosinophilia of unknown significance. The resulting dataset is of high quality and suitable for differential expression analysis. Flow cytometry and qPCR were then performed on three additional cohorts of human subjects, to validate the key findings at the transcript and protein levels. The resulting datasets provide a resource for understanding the response of circulating human eosinophils to glucocorticoid administration.

## Background & Summary

Glucocorticoids effectively suppress eosinophilia and its clinical manifestations, and they are first-line agents in a variety of eosinophil-associated disorders, including hypereosinophilic syndromes^1^ and eosinophilic granulomatosis with polyangiitis^2^. Although commonly used to treat these and other disorders, glucocorticoids have non-specific effects and their use is associated with significant toxicity. Glucocorticoids act primarily through the induction of changes in gene expression. The glucocorticoid receptor is a transcription factor that binds glucocorticoids in the cytoplasm, after which the complex translocates to the nucleus. The ligand-bound glucocorticoid receptor complex then binds genomic DNA directly, or indirectly via tethered interactions with other proteins^3,4^. The glucocorticoid receptor complex also interferes with the activity of other transcription factors, notably NF-κB and AP-1^4^. Although the molecular biology of glucocorticoid receptor signaling has been the subject of intensive study, the specific mechanisms responsible for the clinically beneficial actions of glucocorticoids in different cell types and disease states remain poorly understood^5^. In the case of eosinophils, one of the difficulties in studying the glucocorticoid response *in vivo* is the fact that systemic administration of glucocorticoids consistently leads to a transient but profound drop in the number of circulating eosinophils^6,7^. Consequently, serial sampling of sufficient numbers of eosinophils after glucocorticoid administration in healthy volunteers with normal eosinophil counts (<500/μL) is problematic. Although patients with hypereosinophilic syndromes (HES) have adequate numbers of blood eosinophils for study, glucocorticoid responses in these patients vary considerably and may not reflect normal pathways. Hypereosinophilia of unknown significance (HE_US_) is a rare trait in humans, defined by persistently elevated levels of blood eosinophils (≥1,500/μL for more than 5 years)^8^ and no evidence of clinical manifestations or organ system involvement attributable to the eosinophilia. Moreover, the eosinophil response to glucocorticoids appears to be normal in these subjects. To study the early transcriptional response of human eosinophils to *in vivo* administration of a glucocorticoid, we studied three unrelated subjects with HE_US_ (cohort 1). A single 1 mg/kg oral dose of the glucocorticoid prednisone was administered to each subject. This is a dose of prednisone commonly used in clinical practice. Eosinophils were successfully isolated from peripheral blood drawn prior to and at 30, 60, and 120 min after glucocorticoid administration. Total RNA was extracted from the isolated eosinophils without further *in vitro* manipulation, and high-throughput sequencing (RNA-seq) was performed. Each experimental stage was subjected to thorough and rigorous technical design and validation, leading to the generation of a high-quality RNA-seq dataset that is suitable for differential expression analysis. An initial analysis of the RNA-seq data revealed the induction of a pro-apoptotic transcriptional program and differential expression of key genes related to leukocyte migration^9^. To further evaluate these two observations, we performed three additional sets of experiments:

1. *In vitro* assessment of glucocorticoid-induced changes in gene expression for key eosinophil migration and apoptosis genes (cohort 2). We sampled circulating eosinophils from five donors: four unrelated donors with normal eosinophil counts and one subject with HE_US_. We then exposed the cells *in vitro* to the glucocorticoid dexamethasone (or vehicle, as a negative control) for 30, 60 and 120 min. By qPCR, we evaluated the transcriptional response of three key genes implicated in eosinophil migration (*CXCR4*, *CCR1*, *CCR3*) and seven genes involved in eosinophil apoptosis (*BCL2L11*, *XIAP*, *CASP9*, *PAK1*, *TNFAIP3, NOTCH1*, *ZBTB16*). As a reference gene, we measured expression of the gene encoding the 18S subunit of ribosomal RNA. As a positive control, we measured expression of *TSC22D3*, which is known to increase in expression in response to glucocorticoids in multiple cell types.
2. *In vivo* assessment of apoptosis and cell viability in human eosinophils after glucocorticoid administration (cohort 3). To evaluate whether the induction of a pro-apoptotic transcriptional program led to eosinophil apoptosis *in vivo* prior to the egress of eosinophils from the peripheral circulation, which occurs between 60 and 120 (min) after glucocorticoid administration^9^, we studied a third cohort, comprised of three subjects: two unrelated donors with normal eosinophil counts received a single dose of IV methylprednisolone 250 mg and one patient with HES received a single dose of oral prednisone 1 mg/kg. We then performed flow cytometry for Annexin V and 7-aminoactinomycin D (7-AAD) on circulating eosinophils sampled before and 120 minutes after glucocorticoid administration.
3. *In vitro* assessment of surface expression of key eosinophil migration proteins after glucocorticoid exposure (cohort 4). To assess whether the observation of transcript-level changes on key eosinophil migration genes led to changes in protein abundance at the cell surface within the time frame of glucocorticoid-induced eosinopenia, we studied a fourth cohort, comprised of six unrelated donors with normal eosinophil counts. Peripheral blood leukocytes from each subject were exposed *in vitro* to the glucocorticoid methylprednisolone (or vehicle, as a negative control). We then performed flow cytometry, gating on eosinophils, to evaluate changes in surface expression of the proteins CXCR4, CCR1 and CCR3.

**Figure 1:**
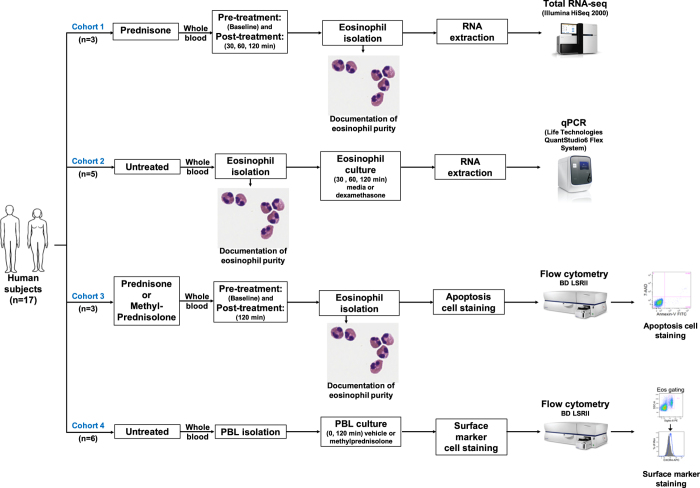
Study design. In cohort 1, three subjects with HE_US_ received a single weight-based dose of prednisone (1 mg/kg). Peripheral blood was collected pre- and post-treatment at 30, 60, and 120 min. Eosinophils were separately isolated from each sample. Eosinophil purity was measured by cytospin preparation stained with eosin and methylene blue. RNA was extracted from isolated eosinophils without further *in vitro* manipulation. Sequencing libraries were separately prepared for each sample, and subjected to RNA-seq. In cohort 2, circulating eosinophils were isolated from five additional unrelated subjects (four donors with normal eosinophil counts and one subject with HE_US_). Purified eosinophils from each subject were cultured with media or 5 μM dexamethasone for 30, 60 and 120 min. RNA was extracted from purified eosinophils without further *in vitro* manipulation and subjected to qPCR. In cohort 3, three unrelated subjects, (two donors with normal eosinophil counts and one patient with HES) were studied. Donors with normal eosinophil counts received a single dose of 250 mg of IV methylprednisolone and the patient with HES received a single weight-based dose of 1 mg/kg of oral prednisone. Peripheral blood was collected pre- and post-treatment at 120 min. Purified eosinophils were stained for apoptosis and cell viability with Annexin-V and 7-AAD, respectively, and analyzed by flow cytometry. In cohort 4, peripheral blood leukocytes (PBL) were isolated from whole blood collected from six unrelated donors with normal eosinophil counts. PBL were cultured with vehicle, 20 μM methylprednisolone or 200 μM methylprednisolone for 120 min. Surface expression of CXCR4, CCR1, and CCR3 was assessed by flow cytometry.

Figure 1 summarizes the study design. We anticipate that the RNA-seq, qPCR, and flow cytometry datasets described here can be used for hypothesis generation in studies of the baseline state of circulating human eosinophils and in studies of the mechanisms behind glucocorticoid-induced eosinopenia and glucocorticoid resistance.

## Methods

These methods are expanded versions of descriptions in our related work^9^.

### Human subjects

Patients with HE_US_ were enrolled under NIH protocol NCT00001406. Patients with HES were enrolled under NIH protocol NCT01524536. Normal donors (ND) were enrolled under NIH protocols NCT02798523, NCT00001846, and NCT000090662. The Institutional Review Board of the National Institute of Allergy and Infectious Diseases at the National Institutes of Health approved each protocol. Informed consent was obtained from each subject prior to enrollment. Demographic information for each subject is provided in Table 1. For the purpose of these studies, a normal donor was defined as an individual without a history of severe allergic reaction to glucocorticoids, autoimmune or autoinflammatory diseases, active solid or hematologic malignancy, diabetes mellitus, cancer chemotherapy within the previous 5 years, surgery within the previous 8 weeks, history of a recent infection (within the previous 30 days), a positive test for human immunodeficiency virus, hepatitis A, B or C virus infection, a history of parasitic, amebic, fungal or mycobacterial infections or other possible latent infections, a history of a bleeding disorder, vaccination within the previous 30 days, a body mass index (BMI) below 18 or above 35, pregnancy, or breastfeeding. Volunteers were not included in the study if they had taken any of the following in the 30 days prior to the screening visit: a glucocorticoid (including topical or inhaled), a nonsteroidal anti-inflammatory drug (including aspirin and selective COX-2 inhibitors), an anti-epileptic drug, an anticoagulant, a statin, a selective serotonin reuptake inhibitor, a macrolide, an azole, diltiazem, troglitazone, rifabutin, ranitidine, rifampin, quinine, quinidine, cyclosporine, amiodarone, St. John’s wort, immunosuppressive or immunomodulatory drugs. Baseline studies included a complete blood count, electrolytes, liver function tests, an interferon gamma release assay for latent tuberculosis infection, and an electrocardiogram. Values outside of the NIH Department of Laboratory Medicine normal reference range and deemed clinically significant by the principal investigator, or any condition that, in the investigator’s opinion, may put the participant at undue risk, were also used as exclusion criteria.

### Blood collection

Peripheral blood was collected in Vacutainer EDTA blood collection tubes (Becton Dickinson, Cat No. 366643). For cohort 1, 10 cc peripheral blood was collected at each time point. For cohort 2, 100 cc peripheral blood was collected from normal donors and 60 cc peripheral blood was collected from the HE_US_ patient. For cohort 3, 30 cc of peripheral blood was collected at each time point from normal donors and 20 cc peripheral blood was collected at each time point from the HES patient. For cohort 4, 30 cc of peripheral blood was collected.

### Eosinophil purification and documentation of purity

At each time point, eosinophils were immediately purified from whole blood by negative-selection immunomagnetic purification with the MACSxpress Eosinophil Isolation Kit followed by the removal of residual erythrocytes with the Erythrocyte Depletion Kit (Miltenyi Biotec, Cat. Nos 130-104-446 and 130-098-196, respectively). The eosinophil fraction was counted, and eosinophil purity was determined by counting a minimum of 300 cells on a cytospin preparation stained with eosin and methylene blue (Kwik-Diff Solution, Thermo Fisher, Cat. No. 9990700). Eosinophil counts from whole-blood samples were obtained with a Siemens ADVIA 120 hematology system.

### RNA extraction for RNA-seq

Eosinophils were isolated from the peripheral blood of each of the three subjects in cohort 1, as described above. Purified eosinophils were then centrifuged at 170×g for 10 min at 4 °C. The resulting pellets were resuspended and homogenized in 500 μL of TRIzol reagent (Thermo Fisher, Cat. No. 15596018) and stored at −80 °C. On the day of RNA extraction, the samples were thawed and allowed to return to room temperature (RT). For every mL of TRIzol, 0.1 mL of the phase separation reagent 1-bromo-3-chloropropane (BCP) (Molecular Research Center, Inc. Cat. No. BP151) was added. The samples were then homogenized by vigorously shaking to an emulsion followed by mixing at RT in an Eppendorf Thermomixer 5436 (Millipore Sigma, Cat. No. Z368164) at 1200 rpm for 15 min. Samples were then centrifuged at 12,000×g for 15 min at 4 °C to separate the phases. The RNA, which is in the upper aqueous phase, was transferred to a new microcentrifuge tube and kept on ice. One back-extraction was performed by adding RNase-free water (one-half of the initial TRIzol volume) to the organic phase, mixing and centrifuging as above. The back-extracted aqueous phase was recovered and pooled with the initial aqueous phase. One volume of 100% ethanol was added to each sample, to precipitate the RNA. This was followed by column-based RNA purification with the RNA Clean & Concentrator-5 kit (Zymo Research; Cat. No. R1016). For this, half the volume of each sample (~600 μL) was transferred to a Zymo-spin IC column. The columns were first centrifuged at 2,000×g for 150 seconds (sec). The flow-through was reloaded on the column, centrifuged at 10,000×g for 30 sec, then discarded. The two centrifugations were repeated for the remaining half of the sample on the respective IC columns. This was followed by one wash with RNA prep buffer and two washes with RNA wash buffer, following the manufacturer’s instructions. Two elutions of 10 μL each, with centrifugation at 10,000 × g for 1 min at RT, were performed with RNase-free water pre-heated to 94 °C. The purified RNA samples were stored at −80 °C.

### RNA sequencing

Sequencing libraries were prepared with the TruSeq Stranded Total RNA with Ribo-Zero Gold Kit (Illumina, Cat. No. RS-122-2303), following the manufacturer’s high-sample (HS) protocol. The input amount of total RNA per sample ranged from 0.4 to 1.5 μg with a mean of 1 μg and a standard deviation of 0.321 μg. The initial step is ribosomal RNA (rRNA) removal from total RNA. The Ribo-Zero Gold reagent depletes samples of both cytoplasmic and mitochondrial rRNA. The next steps are RNA fragmentation and first-strand cDNA synthesis. The latter is carried out in the presence of Actinomycin D, which specifically inhibits DNA-dependent, but not RNA-dependent, DNA synthesis^10^. This is followed by second-strand cDNA synthesis, adenylation of the 3’ ends, adapter ligation, and enrichment of the resulting double-stranded (ds) DNA libraries by 13 cycles of PCR. The sequencing libraries were quantified by a nucleic-acid-binding fluorometric method on a Qubit 2.0 fluorometer (Thermo Fisher, Cat. No. Q32866), with the Qubit dsDNA HS Assay Kit (Thermo Fisher, Cat. No. Q32854). The quality and size distribution of the sequencing libraries were assessed by microfluidic electrophoresis on an Agilent 2100 Bioanalyzer system (Agilent Technologies, Cat. No. G2939A), with DNA 1000 chips (Agilent Technologies, Cat. No. 5067-1504). The enriched libraries had a mean concentration of 57.33 ng/μL with a standard deviation of 4.44. The mode length of the final dsDNA libraries was 300 bp. Based on this estimate, each library was diluted to 2 nM. Libraries were then pooled (11-plex), and the cBot system (Illumina, Cat. No. SY-301-2002) was used for paired-end cluster generation at a concentration of 12 pM, with the TruSeq PE Cluster Kit v3-cBot-HS (Illumina, Cat. No. PE-401-3001). The clustered flow cells underwent paired-end sequencing (2 × 94 bp) on an Illumina HiSeq 2000 sequencer (Illumina, Cat. No. SY-401-1001), with the TruSeq SBS v3-HS kit (Illumina, Cat. No. FC-401-3001).

### Data processing

The output of an Illumina sequencing run is a set of base call (.bcl) files. The bcl files for this dataset were converted to read-level data in FASTQ format with bcl2fastq v.2.17.1.14 (Illumina, Inc.). Adapter sequences were trimmed with Cutadapt v.1.10^11^ in Python v.2.7.9, with the following sequences as input:

Read 1: AGATCGGAAGAGCACACGTCTGAACTCCAGTCAC

Read 2: AGATCGGAAGAGCGTCGTGTAGGGAAAGAGTGT

Adapter-trimmed reads under 20 base-pairs were discarded. The adapter-trimmed FASTQ files were aligned to the reference human genome assembly (GRCh38) with Bowtie v. 2.2.5^12^ and TopHat v.2.0.14^13,14^. Because Bowtie2 is not haplotype-aware, haplotype sequences were excluded from the GRCh38 reference assembly to generate the Bowtie2 genome index file. The transcript annotation (GTF) file was obtained from GENCODE (release 23)^15^ and was also modified to exclude all haplotype sequences prior to generation of the Bowtie2 transcriptome index file. Only fragments in which both paired-end reads were successfully aligned were kept. The binary alignment files (.bam) were then used for generation of a matrix of read counts with the featureCounts program of the package Subread v.1.5.1^16^. Paired-end exonic fragments were grouped at the level of genes, based on the GENCODE 23 annotation file. Normalization was performed with the DESeq2^17^ package in R v.3.3.1^18^. DESeq2 takes as input a matrix of unnormalized read counts, such as the one we generated with featureCounts for this dataset. This matrix (K) has the following format:
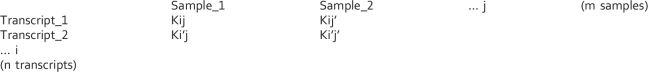


To normalize the read count data, DESeq2 uses the median-of-ratios method. In summary, the read count in transcript *i* from sample *j* is normalized by a factor s_*ij*_ to account for differences in sequencing depth between samples. For this, DESeq2 first obtains a pseudo-reference sample for each transcript, by taking the geometric mean for the transcript across samples (row-wise):

$$K_{i}^{R}={\left(\prod_{j=1}^{m}K_{ij}\right)}^{\frac{1}{m}}$$

It then divides each read-count value by the pseudo-reference sample for its transcript (excluding pseudo-reference sample values of zero), obtaining the following ratio:

$$\frac{K_{ij}}{K_{i}^{R}}$$

For each sample (column-wise), it then calculates the median of the ratios, to get the size factor s_j_. All values within a sample are then divided by the same size factor, so that s_ij_ = s_j_. The resulting values are the normalized read counts. A matrix of normalized read counts for this dataset, obtained as described above, is available as a supplementary file with the GEO upload of this dataset (GSE111789).

### Code availability

Table 2 shows the RNA-seq data processing pipeline, including the versions of all software and the specific variables and parameters used to generate, test, and process the dataset.

### *In vitro* cell culture system for qPCR

Eosinophils were isolated from the peripheral blood of each of the five subjects in cohort 2, as described above. Purified eosinophils were then centrifuged at 170 × g for 10 min at 4 °C. Pelleted cells were resuspended in culture medium consisting of Gibco RPMI (Thermo Fisher, Cat. No. 11835-030) with 10 mM HEPES (Thermo Fisher, Cat. No. 15630-080) at a concentration of 5 × 10^6^ cells/mL. Resuspended cells were then incubated in 12-well flat-bottom plates (Corning, Cat. No. 3513). After resting for 4 hours at 37 °C, 5% CO_2_, 5 μM water-soluble dexamethasone (Sigma-Aldrich, Cat. No. D2915) or vehicle (culture medium) was added, and the cells were incubated for an additional 30, 60 or 120 min. This concentration of dexamethasone is expected to fully saturate the glucocorticoid receptor^19^ and would be approximately equivalent to the peak plasma concentration after a single intravenous dose of 100 mg^20^. At each time point, eosinophils were harvested separately from each well and centrifuged at 170 × g for 10 min at 4 °C. The resulting pellets were individually resuspended and homogenized in 500 μL of TRIzol reagent (Thermo Fisher, Cat. No. 15596018) prior to storage at −80 °C.

### RNA extraction for qPCR

On the day of RNA extraction, the samples were thawed and allowed to return to RT. For every mL of TRIzol, 0.2 mL of the phase separation reagent RNase-free Chloroform (Mallinckrodt Chemicals, Cat. No. 4440-04) was added. The samples were then homogenized by vigorously shaking for 15 sec, incubating for 2 min at RT, and then shaking for 15 sec. Samples were then centrifuged at 10,500×g for 15 min at 4° C to separate the phases. The RNA, which is in the upper aqueous phase, was transferred to a new microcentrifuge tube (Eppendorf, Cat. No. 022363352) and kept on ice. One volume of 100% RNase-Free isopropanol (Fisher Scientific, Cat. No. 9084-01) was added to each sample, vortexed, and incubated  at −20 °C overnight to precipitate the RNA. The following day, the samples were removed from the −20 °C and centrifuged at 10,500×g for 15 min at 4 °C. The supernantants were discarded, and the remaining pellet was washed with one volume of RNase-free 70% ice cold ethanol (Acros Organics, Cat. No. 61509-500) in RNase-free diethyl pyrocarbonate (DEPC)-treated water (Promega, Cat. No. P1195). The pellets were briefly vortexed and then centrifuged at 10,500 × g for 10 min at 4°C. The supernantants were discarded and the remaining pellet was allowed to dry for 5 min upside down. The pellets were resuspended with 15 μl of RNase-free DEPC-treated water to re-dissolve RNA, vortexed for 30 s, then held at 4 °C. The RNA concentration and purity were measured with a spectrophotometer (DeNovix, Cat. No. DS-11) prior to storage of RNA samples at −80 °C.

### qPCR

cDNA was synthesized from isolated, cultured eosinophil RNA with the High Capacity cDNA Reverse Transcription Kit (Applied Biosystems, Cat. No. 4374967), RNase Inhibitor (Applied Biosystems, Cat. No. N8080119) and GeneAmp dNTP blend (Applied Biosystems, Cat. No. N8080260). 1 μg RNA was prepared per 100 μL reaction in RNase-free microcentrifuge tubes. The RNA samples were placed in a thermal cycler (Applied Biosystems, Veriti 96-well thermal cycler, Cat. No. 4375786) using a custom program (25 °C for 10 min, 37 °C for 60 min, 95 °C for 5 min, and 4 °C ∞) to synthesize cDNA. Quantitative real-time PCR was performed in duplicate using the QuantStudio6 Flex System (Applied Biosystems, Cat. No. 278861532) with the TaqMan Fast Universal PCR Master Mix (Applied Biosystems, Cat No. 4352042), and FAM-labeled TaqMan gene expression assay sets for *CXCR4*, *CCR1*, *XIAP*, *CCR3*, *NOTCH1*, *ZBTB16*, *PAK1*, *CASP9*, *TNFAIP3*, *BCL2L11* and *TSC22D3* (Applied Biosystems, Cat. Nos Hs00237052_m1, Hs00174298_m1, Hs02837743_m1, Hs04931116_m1, Hs01062014_m1, Hs00232313_m1, Hs00945621_m1, Hs00962278_m1, Hs00234713_m1, Hs01076940_m1 and Hs00608272_m1, respectively) or the VIC-labeled eukaryotic 18S rRNA endogenous control (Applied Biosystems, Cat. No. 4318839). For qPCR, DNase treatment was not performed because Taqman primer/probe sets were designed to span at least one intron-exon boundary; therefore, DNA containing introns was excluded. qPCR was conducted with a custom program (increase at a rate of 1.6 °C/sec from 25°C to 95 °C, hold at 95 °C for 20 s, decrease at a rate of 1.6 °C/sec to 60 °C, hold at 60 °C for 20 s and then increase to 95 °C at 1.9 °C/sec to complete cycle), and the mean Ct values for each subject, in each condition (dexamethasone or medium), at each time point, were calculated as the mean of two technical replicates.

### Analysis of eosinophil apoptosis and viability by flow cytometry

Eosinophils were isolated from the peripheral blood of each of the three subjects in cohort 3, as described above. Purified eosinophils were resuspended in freshly prepared 1X binding buffer (Becton Dickinson, Cat. No. 556454) and stained at room temperature for 15 min with 5 μL Annexin-V FITC (fluorescein isothiocyanate, Becton Dickenson, Cat. No. 556419) and 5 μL 7-AAD (7-aminoactinomycin D, Becton Dickenson, Cat. No. 559925). After the addition of 100 μL of 1X PBS (Quality Biological, Cat. No. 114-058-101), signal acquisition was immediately performed on a Becton Dickinson LSR II flow cytometer at the Flow Cytometry Section of the Research Technologies Branch at the National Insitute of Allergy and Infectious Diseases (NIAID).

### Analysis of eosinophil cell-surface proteins by flow cytometry

Each blood sample was diluted 1:10 in ACK lysis buffer (Quality Biological, Cat. No.118-156-101). Samples were then incubated for 10 min at RT with shaking to lyse the erythrocytes. The remaining cells (peripheral blood leukocytes, PBL) were pelleted by centrifugation at 300 x g, 4 °C, for 5 min. PBLs were then resuspended at a concentration of 4 million cells/mL in culture medium consisting of Gibco RPMI (Thermo Fisher, Cat. No. 11835-030) supplemented with 10 mM HEPES (Thermo Fisher, Cat. No. 15630-080). 250 μL of cell suspension were added per well to 48-well flat-bottom plates (Costar, Cat. No. 3548). After resting for 2 hours at 37 °C with 5% CO_2_, a fraction of the cells was sampled for cell surface expression (baseline). The remaining PBLs were incubated for an additional 120 min with media alone, media with vehicle (0.1% ethanol), media with vehicle and 20 μg/dL methylprednisolone (MP) (Sigma-Aldrich, Cat. No. M0639), or media with vehicle and 200 μg/dL MP. These concentrations of methylprednisolone approximately correspond to the expected peak plasma concentrations after a single intravenous dose of 25 mg or 250 mg, respectively^21^. Surface marker staining was performed before (baseline) and after (120 min) *in vitro* exposure of PBLs to MP. Cell suspensions were pelleted by centrifugation at 300×g, 4 °C for 5 min. Cell pellets were resuspended in 100 μL 1X PBS (Quality Biological, Cat. No. 114-058-101), 1% BSA (MP Biomedicals, Cat. No. 0216006990) and 4% mouse serum (Sigma Aldrich, Cat. No. M5905) and blocked for 10 min at 4 °C. Blocked cells were then stained with the following antibodies: CD45-PerCP, clone 2D1 (BD Biosciences, Cat. No. 347464), Siglec-8-PE, clone 7C9 (Biolegend, Cat. No. 347104), CCR1-Alexa Fluor 488, clone 53504 (R&D Systems, Cat. No. FAB145G), CCR3-APC-Cy7, clone 5E8 (Biolegend, Cat. No. 310712), and CXCR4-APC, clone 12G5 (BD Biosciences, Cat. No. 555976). Isotype controls were used to ensure non-specific staining did not occur. The following isotype controls (ITCL) were used: Mouse IgG1 Kappa ITCL PerCP (BD Biosciences, Cat. No. 550672), Mouse IgG1 Kappa ITCL PE (Biolegend, Cat No. 400112), Mouse IgG2b ITCL Alexa Fluor 488 (R&D Systems, Cat. No. IC0041G), Mouse IgG2b Kappa ITCL APC-Cy7 (Biolegend, Cat. No. 400328) and Mouse IgG2a Kappa ITCL APC (BD Biosciences, Cat. No. 555576). Signal acquisition was performed on Becton Dickinson LSR II flow cytometer at the Flow Cytometry Section of the NIAID Research Technologies Branch.

## Data Records

The RNA-seq dataset is deposited in Gene Expression Omnibus (GEO) under series number GSE111789 (Data Citation 1). The GEO entry includes links to the raw data in FASTQ format, which is deposited in the Sequence Read Archive (SRA) under SRP135489 (Data Citation 2). A file with processed data is provided with the GEO series record. The values correspond to read counts normalized by library size. The file contains a table with 58,765 rows and 13 columns. The first row is a header row, and it is followed by 58,764 rows, each corresponding to one transcript. The first column has the transcript identifiers and it is followed by 12 columns, one for each sample. Table 3 provides a description of each sample and its respective GEO sample accession (GSM) number. The qPCR dataset is deposited in the figshare database (Data Citation 3). Table 4 provides a description of each sample. The apoptosis flow cytometry experiment files for cohort 3 each contain five or six .fcs files corresponding to each condition (unstained or stained) at each time point (baseline, 60 minutes and/or 120 minutes). Table 5 provides a description of each sample and its respective .fcs file name as uploaded to FlowRepository under Repository ID FR-FCM-ZYNE (Data Citation 4). The surface marker flow cytometry experiment files for cohort 4 each contain seven or eight .fcs files corresponding to each condition (unstained or stained) at each time point (baseline or 120 minutes) with each treatment (none, vehicle, 20 μg/dL methylprednisolone, and 200 μg/dL methylprednisolone). Table 6 provides a description of each sample and its respective .fcs file as uploaded to FlowRepository under Repository ID FR-FCM-ZYND (Data Citation 5).

## Technical Validation

### Eosinophil purity

Eosinophil purity was defined as the proportion of eosinophils among all counted leukocytes on a cytospin preparation. A minimum eosinophil purity of 98% was documented in all samples.

### RNA quality control

**Figure 2:**
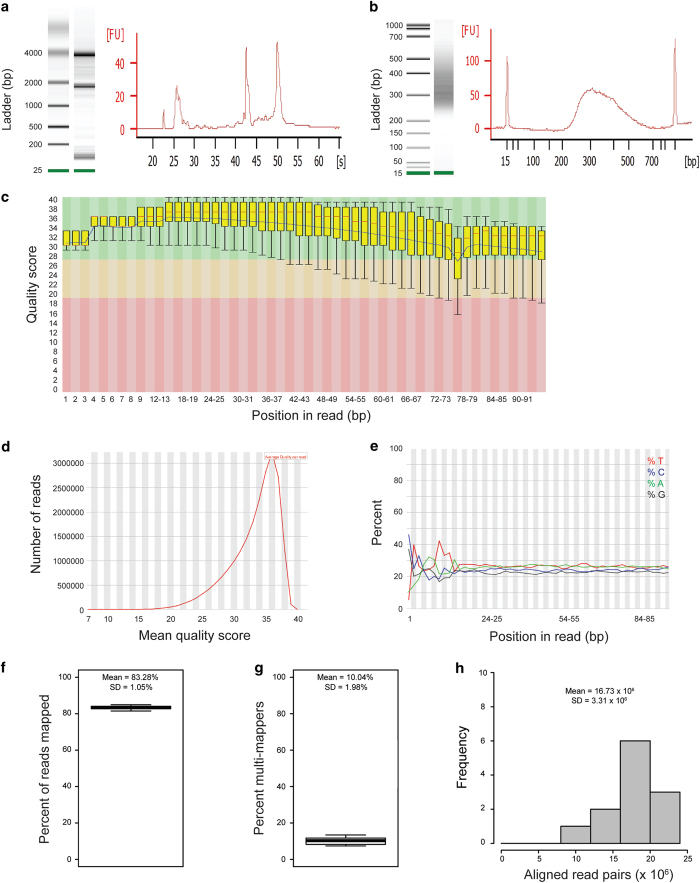
Quality control of the RNA samples, sequencing libraries, sequencing reads, and read alignments. (**a**) Electropherogram of a representative total RNA sample from this study, following extraction and prior to library preparation. The sample displayed had an RNA integrity number (RIN) of 9, which was the mean for all samples in the study. (**b**) Electropherogram of a representative dsDNA sequencing library from this study. The size distribution of the dsDNA molecules was very similar for all the libraries, with a mode around 300 bp. (**c**) Distribution of quality scores by base pair for a representative FASTQ file from this study. The quality scores on the y-axis are defined as -10log_10_*e*, where *e* is the estimated probability of a base call being wrong. Therefore, a quality score of 30 means that the estimated probability of a base being wrong is 1/1000. For each position in the sequenced reads, the corresponding box plot displays the distribution of quality scores across all the sequences in a FASTQ file. In each box plot, the red line displays the median, the yellow box the interquartile range (25–75%), and the lower and upper whiskers the 10^th^ and 90^th^ percentiles, respectively. The blue line displays the mean quality scores. **(d**) Distribution of the mean quality score by read for a representative FASTQ file from this study. (**e**) Sequence content in a representative FASTQ file from this study. For each position in the sequenced reads, the sequence content is the proportion of each of the four nucleotides (A, T, C, and G) at that position. (**f**) Distribution of mapping rates for the 12 samples in this study. The mapping rate is defined as the percent of reads in each sample that were uniquely aligned to the reference genome. (**g**) Distribution of the percentage of multi-mappers for the 12 samples in this study. This represents the percentage of mapped reads that aligned to more than one location in the reference genome. (**h**) Distribution of the number of aligned read pairs for the 12 samples in this study. Each read pair is counted once.

Quality of the isolated RNA samples for RNA-seq was assessed by microfluidic electrophoresis on an Agilent 2100 Bioanalyzer system (Agilent, Cat. No. G2939A), with RNA 6000 Nano chips (Agilent, Cat. No. 5067-1511). Each electropherogram was manually reviewed. As a second measure of quality, we used the RNA integrity number (RIN), which is calculated by a proprietary algorithm of Agilent Technologies. This algorithm was developed based on manual grading of electropherograms and machine learning, and it offers a quantitative assessment of RNA quality. This assessment is based on a numbering system from 1 to 10, with 1 being the most degraded profile and 10 being the most intact. The total RNA samples in this dataset had a mean RIN of 9 with a standard deviation of 0.439. A representative sample from this study, with a RIN of 9, is presented in Fig. 2a. RNA quantity was measured on a Qubit 2.0 fluorometer (Thermo Fisher, Cat. No. Q32866), with RNA BR quantitation assays (Thermo Fisher, Cat. No. Q10211). All of the purified RNA samples had concentrations in the range of 80 to 500 ng/μL, with a mean of 266 ng/μL and standard deviation of 130 ng/μL. Quality of the isolated RNA samples for qPCR was assessed by measuring the A260/280 ratio by spectrophotometry. The mean of 20 of the 26 RNA samples A260/280 ratio was 1.666 with a standard deviation of 0.219 and a range of 1.441 to 2.101. A260/280 was not measured for 6 samples. All of the purified RNA samples had concentrations in the range of 5 to 509 ng/μL, with a mean of 122 ng/μL and standard deviation of 128 ng/μL.

### RNA-seq library validation

The size distribution and quality of the dsDNA sequencing libraries were analyzed by microfluidic electrophoresis on an Agilent Technologies 2100 Bioanalyzer (Agilent, Cat. No. G2939A), with DNA 1000 chips (Agilent, Cat. No. 5067-1504). The size distribution was consistent for all the samples, with a mode length of 300 bp. A representative library from this study is displayed in Fig. 2b. The mean library concentration was 57.33 ng/μL with a standard deviation of 4.44, as quantified on a Qubit 2.0 fluorometer (Thermo Fisher, Cat. No. Q32866), with dsDNA HS Assay Kits (Thermo Fisher, Cat. No. Q32854).

### Quality control of the sequencing reads

We performed quality control of the sequencing read files for this dataset (in FASTQ format), after adapter-sequence trimming, with the software FastQC (https://www.bioinformatics.babraham.ac.uk/projects/fastqc). We are not aware of a single specific criterion that reliably measures the quality of a FASTQ file. However, FastqQC provides several data displays that are useful for identifying outlier samples. We relied primarily on the following three:

- The distribution of quality scores for each base across all reads in file (a representative plot from this dataset is in Fig. 2c).
- The distribution of the mean quality scores across all reads in a file (a representative plot from this dataset is in Fig. 2d).
- The sequence content (proportion of each of the four nucleotides A, T, C, and G) for each base across all reads in a file (a representative plot from this dataset is in Fig. 2e).

In the example displayed in Fig. 2c, which is representative of this dataset, only one base position had an interquartile range below 28, indicating that at least 75% of the reads have quality scores above 28, so that the probability of a base call being wrong is no more than 0.0016. Figure 2d shows a mode quality score of 35, indicating on average a very low probability (around 0.0003) of a base call being wrong. Based on this type of analysis, the sequencing reads for all FASTQ files were of high quality, and we did not identify any outlier samples in the dataset.

### Alignment statistics

The distribution of the proportion of all adapter-trimmed reads that were successfully aligned (mapped) to the reference genome among the 12 samples in this study is presented in Fig. 2f. This percentage of aligned reads (referred in TopHat2 as the overall mapping rate) had a mean of 83.23% with a standard deviation of 1.05%. Of the aligned reads from each sample, the proportion of reads that did not align uniquely to the reference genome (multi-mappers) had a mean of 10% and a standard deviation of 1.98%. The distribution of the percentage of multi-mappers in this study is presented in Fig. 2g. The total number of aligned read pairs (where each read pair is counted once) had a mean of 16,730,000 and a standard deviation of 3,310,000. The distribution of aligned read pairs for the 12 samples in this study is presented in Fig. 2h.

### Correlation among biological replicates

**Figure 3:**
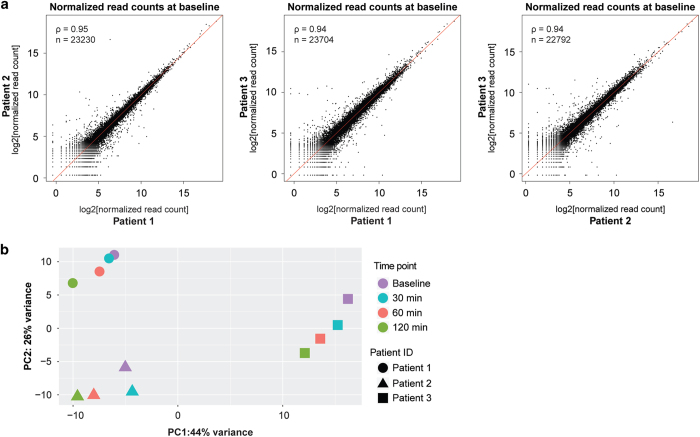
Quality control of the RNA-seq replicates. (**a**) The plots display log2-transformed normalized read count values. Each dot represents one transcript. Transcripts with no evidence of expression in either subject (read count of 0) were excluded. The number of transcripts with non-zero read counts in each pairwise comparison is denoted by n. The Spearman rank correlation coefficient for each pairwise comparison is denoted by rho. (**b**) Principal Component Analysis (PCA) plot of the 12 samples. Regularized log-transformed read counts were used as input and the samples are spanned in the two-dimensional plane by their first two principal components. The proportion of the variance explained by each component is displayed in the respective axis.

To assess the level of similarity in the baseline transcriptome of circulating eosinophils from the three unrelated patients studied, we plotted the normalized read count values at baseline for all expressed transcripts in each of the three pairwise comparisons (Fig. 3a). An expressed transcript was defined as a transcript with a read count > 0 in at least one of the two samples being compared. The baseline eosinophil transcriptomes of the three unrelated patients were similar, with Spearman’s rank correlation coefficients of 0.94 to 0.95 (Fig. 3a).

To evaluate whether the overall change in the eosinophil transcriptome induced by the glucocorticoid stimulus was similar in the three patients at the three post-treatment sampling times, we performed principal component analysis (PCA) on the count data at each time point. For this, we first obtained data in the log scale normalized for library size, by applying the regularized log transformation (rlog) method as implemented in DESeq2^17^. This method avoids a common property of the standard logarithm transformation, which is the spreading apart of data for genes with low counts. The rlog transformation behaves similarly to a log2 transformation for genes with high counts but shrinks the values for genes with low counts. In short, this method fits a model:

$$\log_{2}\left(q_{i,j}\right)=\beta_{i,0}+\beta_{i,j}$$

Where *q*_*i,j*_ is a parameter proportional to the true read count of transcript *i* in sample *j*, *β*_*i*,0_ is an intercept which does not undergo shrinkage, and *β*_*i,j*_ is a sample-specific effect, which is shrunk towards zero based on the trend of the dispersion across read-count values in the samples. The goal of performing this transformation prior to PCA analysis is to render the data homoscedastic. The PCA plot of the rlog-transformed data for the 12 samples in this study is presented in Fig. 3b. All patients showed a decrease with respect to PC2 over time and a decrease with respect to PC1, except for a slight increase in patient 2 from baseline to 30 min. This suggests that the overall effect of the glucocorticoid over time in the eosinophil transcriptome was similar in the three patients studied.

### Quality control of qPCR data

A no-template control was used to verify no signal was produced from the reagents with water alone. The gene encoding the 18S subunit of ribosomal RNA was used as a reference gene to normalize all qPCR results. For qPCR, Taqman primer/probe sets were designed to span at least one intron-exon boundary; therefore, DNA containing introns was excluded. The gene *TSC22D3*, which is known to increase in response to glucocortiocids in multiple cell types, was included as a positive control in qPCR experiments. The expected increase in *TSC22D3* expression was observed in all glucocorticoid-treated samples and in none of the vehicle-treated samples.

### Quality control of the flow cytometry data

Autofluorescence in each sample set was established with an unstained control. Single-color controls using PBL at baseline stained with each fluorophore were used to account for spillover and assign a compensation matrix to the samples. Fluorescence minus one (FMO) controls were used to assign gates. Isotype controls were used as negative controls to ensure that non-specific staining did not occur. All isotype controls were negative for their corresponding fluorochrome.

## Usage Notes

GEO requests the upload of untrimmed FASTQ files, which are made publicly available through SRA. Therefore, the publicly-available raw data files for this study have untrimmed, 2 × 94 bp reads. As described in the Methods section and in Table 2, we chose to trim adapter sequences prior to alignment. We recommend that investigators using our data, particularly those trying to reproduce our results, to do the same.

## Additional information

**How to cite this article**: Gadkari, M. *et al*. Transcript- and protein-level analyses of the response of human eosinophils to glucocorticoids. *Sci. Data*. 5:180275 doi: 10.1038/sdata.2018.275 (2018).

**Publisher’s note**: Springer Nature remains neutral with regard to jurisdictional claims in published maps and institutional affiliations.

## Supplementary Material



Supplementary File 1

## Figures and Tables

**Table 1:** Demographic information.

Cohort	ID	Diagnosis	Age (y)	Gender	Race	Weight (kg)	Baseline AEC	Assay
1	Patient_1	HE_US_	34	M	White	92	2180	RNA-seq
1	Patient_2	HE_US_	69	M	White	68.1	2060	RNA-seq
1	Patient_3	HE_US_	35	F	White	73.9	3340	RNA-seq
2	Patient_4	ND	33	F	White	NA	200	qPCR
2	Patient_5	ND	46	M	White	NA	151	qPCR
2	Patient_6	ND	33	M	Asian	NA	170	qPCR
2	Patient_7	HE_US_	35	M	White	NA	1470	qPCR
2	Patient_8	ND	29	M	White	NA	380	qPCR
3	Patient_9	HES	45	F	White	87.6	1660	Flow cytometry (apoptosis)
3	Patient_10	ND	47	M	African American	119.3	120	Flow cytometry (apoptosis)
3	Patient_11	ND	25	M	Asian	95.2	260	Flow cytometry (apoptosis)
4	Patient_12	ND	64	M	White	NA	70	Flow cytometry (surface markers)
4	Patient_13	ND	31	M	White	NA	410	Flow cytometry (surface markers)
4	Patient_14	ND	50	F	Asian	NA	140	Flow cytometry (surface markers)
4	Patient_15	ND	25	M	Asian	NA	200	Flow cytometry (surface markers)
4	Patient_16	ND	39	F	White	NA	240	Flow cytometry (surface markers)
4	Patient_17	ND	47	M	White	NA	610	Flow cytometry (surface markers)
Subject identifiers in the ID column correspond to those in the public repositories (GEO, figshare, and FlowRepository for RNA-seq, flow cytometry, and qPCR datasets, respectively). AEC: Absolute eosinophil count in peripheral circulation (eosinophils/μL). HEUS: Hypereosinophilia of unknown significance. HES: Hypereosinophilic syndrome. ND: Normal donor.								

**Table 2:** RNA-seq data processing pipeline.

Data processing step	Software/version	Specific variables and parameters
bcl conversion	bcl2fastq v. 2.17.1.14	
Adapter-sequence trimming	Cutadapt v. 1.10 on Python v. 2.7.9.	Adapter sequences:
		Read1: AGATCGGAAGAGCACACGTCTGAACTCCAGTCAC
		Read2: AGATCGGAAGAGCGTCGTGTAGGGAAAGAGTGT. Adapter-trimmed reads under 20 base-pairs were discarded.
Alignment	Bowtie2 v. 2.2.5 and TopHat v. 2.0.14	TopHat parameters: --no-mixed --mate-inner-dist 0 --mate-std-dev 50 -p 8 --library-type fr-firststrand
Read counting	featureCounts (Subread v. 1.5.1)	featureCounts parameters: -p -B -s 2 -T 8 -O -t exon -g gene_name
Normalization and differential expression	R package DESeq2 v. 1.16.1, in R v. 3.4.0	

**Table 3:** Description of the RNA-seq data uploaded to Gene Expression Omnibus (GEO).

Cohort	ID	Time point	Treatment	Cell Type	Molecular Extraction	Assay	Accession
1	Patient_1	Baseline	None	Eosinophils	RNA extraction	RNA-seq	GSM3039712
1	Patient_1	30 min	Prednisone	Eosinophils	RNA extraction	RNA-seq	GSM3039713
1	Patient_1	60 min	Prednisone	Eosinophils	RNA extraction	RNA-seq	GSM3039714
1	Patient_1	120 min	Prednisone	Eosinophils	RNA extraction	RNA-seq	GSM3039715
1	Patient_2	Baseline	None	Eosinophils	RNA extraction	RNA-seq	GSM3039716
1	Patient_2	30 min	Prednisone	Eosinophils	RNA extraction	RNA-seq	GSM3039717
1	Patient_2	60 min	Prednisone	Eosinophils	RNA extraction	RNA-seq	GSM3039718
1	Patient_2	120 min	Prednisone	Eosinophils	RNA extraction	RNA-seq	GSM3039719
1	Patient_3	Baseline	None	Eosinophils	RNA extraction	RNA-seq	GSM3039720
1	Patient_3	30 min	Prednisone	Eosinophils	RNA extraction	RNA-seq	GSM3039721
1	Patient_3	60 min	Prednisone	Eosinophils	RNA extraction	RNA-seq	GSM3039722
1	Patient_3	120 min	Prednisone	Eosinophils	RNA extraction	RNA-seq	GSM3039723
Data are deposited under GEO series GSE111789.							

**Table 4:** Description of the qPCR data uploaded to the figshare database.

Cohort	ID	Time point	Treatment	Cell Type	Molecular Extraction	Assay
2	Patient_4	30 min	Media	Eosinophils	RNA extraction	qPCR
2	Patient_4	30 min	Dexamethasone	Eosinophils	RNA extraction	qPCR
2	Patient_4	60 min	Media	Eosinophils	RNA extraction	qPCR
2	Patient_4	60 min	Dexamethasone	Eosinophils	RNA extraction	qPCR
2	Patient_4	120 min	Media	Eosinophils	RNA extraction	qPCR
2	Patient_4	120 min	Dexamethasone	Eosinophils	RNA extraction	qPCR
2	Patient_5	60 min	Media	Eosinophils	RNA extraction	qPCR
2	Patient_5	60 min	Dexamethasone	Eosinophils	RNA extraction	qPCR
2	Patient_5	120 min	Media	Eosinophils	RNA extraction	qPCR
2	Patient_5	120 min	Dexamethasone	Eosinophils	RNA extraction	qPCR
2	Patient_6	30 min	Media	Eosinophils	RNA extraction	qPCR
2	Patient_6	30 min	Dexamethasone	Eosinophils	RNA extraction	qPCR
2	Patient_6	60 min	Media	Eosinophils	RNA extraction	qPCR
2	Patient_6	60 min	Dexamethasone	Eosinophils	RNA extraction	qPCR
2	Patient_6	120 min	Media	Eosinophils	RNA extraction	qPCR
2	Patient_6	120 min	Dexamethasone	Eosinophils	RNA extraction	qPCR
2	Patient_7	30 min	Media	Eosinophils	RNA extraction	qPCR
2	Patient_7	30 min	Dexamethasone	Eosinophils	RNA extraction	qPCR
2	Patient_7	60 min	Media	Eosinophils	RNA extraction	qPCR
2	Patient_7	60 min	Dexamethasone	Eosinophils	RNA extraction	qPCR
2	Patient_7	120 min	Media	Eosinophils	RNA extraction	qPCR
2	Patient_7	120 min	Dexamethasone	Eosinophils	RNA extraction	qPCR
2	Patient_8	60 min	Media	Eosinophils	RNA extraction	qPCR
2	Patient_8	60 min	Dexamethasone	Eosinophils	RNA extraction	qPCR
2	Patient_8	120 min	Media	Eosinophils	RNA extraction	qPCR
2	Patient_8	120 min	Dexamethasone	Eosinophils	RNA extraction	qPCR
For each time point, results are provided for each of the following TaqMan Gene Expression Assays: CXCR4, CCR1, XIAP, CCR3, NOTCH1, ZBTB16, PAK1, CASP9, TNFAIP3, BCL2L11 and TSC22D3.						

**Table 5:** Description of the eosinophil apoptosis and viability data uploaded to FlowRepository.

Cohort	ID	Time point	Treatment	Cell Type	Assay	FCS File
3	Patient_9	Baseline	None	Eosinophils	Flow cytometry (apoptosis)	Patient_9_Baseline_None.fcs
3	Patient_9	120 min	Prednisone	Eosinophils	Flow cytometry (apoptosis)	Patient_9_120min_Prednisone.fcs
3	Patient_10	Baseline	None	Eosinophils	Flow cytometry (apoptosis)	Patient_10_Baseline_None.fcs
3	Patient_10	60 min	MP	Eosinophils	Flow cytometry (apoptosis)	Patient_10_60min_MP.fcs
3	Patient_10	120 min	MP	Eosinophils	Flow cytometry (apoptosis)	Patient_10_120min_MP.fcs
3	Patient_11	Baseline	None	Eosinophils	Flow cytometry (apoptosis)	Patient_11_Baseline_None.fcs
3	Patient_11	60 min	MP	Eosinophils	Flow cytometry (apoptosis)	Patient_11_60min_MP.fcs
3	Patient_11	120 min	MP	Eosinophils	Flow cytometry (apoptosis)	Patient_11_120min_MP.fcs
Data are deposited under Repository ID FR-FCM-ZYNE. MP: methylprednisolone.						

**Table 6:** Description of the flow cytometry data on eosinophil CCR1, CCR3 and CXCR4 surface expression in response to glucocorticoids, uploaded to FlowRepository.

Cohort	ID	Time point	Treatment	Cell Type	Assay	FCS File
4	Patient_12	Baseline	None	PBL	Flow cytometry (surface markers)	Patient_12_Baseline_None.fcs
4	Patient_12	120 min	VH	PBL	Flow cytometry (surface markers)	Patient_12_120min_VH.fcs
4	Patient_12	120 min	20 mg/dL MP	PBL	Flow cytometry (surface markers)	Patient_12_120min_MP20.fcs
4	Patient_12	120 min	200 mg/dL MP	PBL	Flow cytometry (surface markers)	Patient_12_120min_MP200.fcs
4	Patient_13	Baseline	None	PBL	Flow cytometry (surface markers)	Patient_13_Baseline_None.fcs
4	Patient_13	120 min	VH	PBL	Flow cytometry (surface markers)	Patient_13_120min_VH.fcs
4	Patient_13	120 min	20 mg/dL MP	PBL	Flow cytometry (surface markers)	Patient_13_120min_MP20.fcs
4	Patient_13	120 min	200 mg/dL MP	PBL	Flow cytometry (surface markers)	Patient_13_120min_MP200.fcs
4	Patient_14	Baseline	None	PBL	Flow cytometry (surface markers)	Patient_14_Baseline_None.fcs
4	Patient_14	120 min	VH	PBL	Flow cytometry (surface markers)	Patient_14_120min_VH.fcs
4	Patient_14	120 min	20 mg/dL MP	PBL	Flow cytometry (surface markers)	Patient_14_120min_MP20.fcs
4	Patient_14	120 min	200 mg/dL MP	PBL	Flow cytometry (surface markers)	Patient_14_120min_MP200.fcs
4	Patient_15	Baseline	None	PBL	Flow cytometry (surface markers)	Patient_15_Baseline_None.fcs
4	Patient_15	120 min	VH	PBL	Flow cytometry (surface markers)	Patient_15_120min_VH.fcs
4	Patient_15	120 min	20 mg/dL MP	PBL	Flow cytometry (surface markers)	Patient_15_120min_MP20.fcs
4	Patient_15	120 min	200 mg/dL MP	PBL	Flow cytometry (surface markers)	Patient_15_120min_MP200.fcs
4	Patient_16	Baseline	None	PBL	Flow cytometry (surface markers)	Patient_16_Baseline_None.fcs
4	Patient_16	120 min	VH	PBL	Flow cytometry (surface markers)	Patient_16_120min_VH.fcs
4	Patient_16	120 min	20 mg/dL MP	PBL	Flow cytometry (surface markers)	Patient_16_120min_MP20.fcs
4	Patient_16	120 min	200 mg mg/dL MP	PBL	Flow cytometry (surface markers)	Patient_16_120min_MP200.fcs
4	Patient_17	Baseline	None	PBL	Flow cytometry (surface markers)	Patient_17_Baseline_None.fcs
4	Patient_17	120 min	VH	PBL	Flow cytometry (surface markers)	Patient_17_120min_VH.fcs
4	Patient_17	120 min	20 mg/dL MP	PBL	Flow cytometry (surface markers)	Patient_17_120min_MP20.fcs
4	Patient_17	120 min	200 mg/dL MP	PBL	Flow cytometry (surface markers)	Patient_17_120min_MP200.fcs
Data are deposited under Repository ID FR-FCM-ZYND. VH: vehicle; MP: methylprednisolone, PBL: peripheral blood leukocytes.						
